# MiR-7-5p Is Involved in Ferroptosis Signaling and Radioresistance Thru the Generation of ROS in Radioresistant HeLa and SAS Cell Lines

**DOI:** 10.3390/ijms22158300

**Published:** 2021-08-02

**Authors:** Kazuo Tomita, Taisuke Nagasawa, Yoshikazu Kuwahara, Seiji Torii, Kento Igarashi, Mehryar Habibi Roudkenar, Amaneh Mohammadi Roushandeh, Akihiro Kurimasa, Tomoaki Sato

**Affiliations:** 1 Department of Applied Pharmacology, Kagoshima University Graduate School of Medical and Dental Sciences, Kagoshima University, Kagoshima-City 890-8544, Kagoshima, Japan; nagasawa@d1.dent.kagoshima-u.ac.jp (T.N.); y-kuwahara@tohoku-mpu.ac.jp (Y.K.); knt-igrs@dent.kagoshima-u.ac.jp (K.I.); roudkenar@gums.ac.ir (M.H.R.); mohammadi_roushandeh@gums.ac.ir (A.M.R.); tomsato@dent.kagoshima-u.ac.jp (T.S.); 2 Division of Radiation Biology and Medicine, Faculty of Medicine, Tohoku Medical and Pharmaceutical University, Sendai-City 983-8536, Miyagi, Japan; kurimasa@tohoku-mpu.ac.jp; 3 Center for Food Science and Wellness, Gunma University, Maebashi-City 371-8510, Gunma, Japan; storii@gunma-u.ac.jp; 4 Burn and Regenerative Medicine Research Center, Velayat Hospital, School of Medicine, Guilan University of Medical Sciences, Rasht 41937-13194, Iran

**Keywords:** microRNA, reactive oxygen species (ROS), clinically relevant radioresistant (CRR) cells, ferroptosis, Fe^2+^, *ALOX12*

## Abstract

In cancer therapy, radioresistance or chemoresistance cells are major problems. We established clinically relevant radioresistant (CRR) cells that can survive over 30 days after 2 Gy/day X-ray exposures. These cells also show resistance to anticancer agents and hydrogen peroxide (H_2_O_2_). We have previously demonstrated that all the CRR cells examined had up-regulated miR-7-5p and after miR-7-5p knockdown, they lost radioresistance. However, the mechanism of losing radioresistance remains to be elucidated. Therefore, we investigated the role of miR-7-5p in radioresistance by knockdown of miR-7-5p using CRR cells. As a result, knockdown of miR-7-5p increased reactive oxygen species (ROS), mitochondrial membrane potential, and intracellular Fe^2+^ amount. Furthermore, miR-7-5p knockdown results in the down-regulation of the iron storage gene expression such as ferritin, up-regulation of the ferroptosis marker *ALOX12* gene expression, and increases of Liperfluo amount. H_2_O_2_ treatment after *ALOX12* overexpression led to the enhancement of intracellular H_2_O_2_ amount and lipid peroxidation. By contrast, miR-7-5p knockdown seemed not to be involved in *COX-2* and glycolysis signaling but affected the morphology of CRR cells. These results indicate that miR-7-5p control radioresistance via ROS generation that leads to ferroptosis.

## 1. Introduction

Reactive oxygen species (ROS) function as an important mediator in intracellular signal transduction pathways [1]. ROS are generated as a by-product of normal aerobic respiration and several other cellular enzymatic reactions. However, elevated and sustained ROS production or exposure causes oxidative stress, inducing cell death and initiating cancer [2]. ROS are also used for anticancer therapy because ROS can kill cells. In radiation and chemotherapy, such as bleomycin treatment, intracellular ROS levels are elevated and apoptosis is induced in cancer cells [3,4]. However, in radiation or chemotherapy, radioresistance or chemoresistance cells (resistant cells) that produce less ROS are obstacles to overcoming cancer. Many studies have been conducted to overcome the resistant cells. For example, the *COX-2* reported to be controlled by mitochondrial ROS (mtROS) is involved in the resistance to radiotherapy and chemotherapy [5]. Additionally, *COX-2* promotes apoptotic resistance via G1 phase delay, bcl2 induction, and caspase-3 suppression [6,7,8]. It has also been reported that the increased glycolysis results in chemoresistance and radioresistance [9,10]. Increased glycolysis inactivates the mitochondria, the organelle where ATP synthesis by oxidative phosphorylation and ROS production occurs. However, most studies were conducted with cell lines from different genomic backgrounds to understand the characteristics of the resistant cells [11,12,13,14]. Therefore, we have established clinically relevant radioresistant (CRR) cells that can survive over 30 days after 2 Gy/day X-ray exposures and have a similar genomic background [15,16,17].

CRR cells show resistance against irradiation and anticancer agents such as docetaxel. CRR cells also show resistance against ROS such as hydrogen peroxide (H_2_O_2_) [16,18]. CRR cells also have low lipid peroxidation, mitochondrial membrane potential (ΔΨm), mtDNA copy numbers, and ATP amount [16,18]. There is a strong relationship between mtROS and ΔΨm [19]. In fact, mtROS decreased in CRR cells [16]. Recently, it has been reported that miR-7-5p expression is up-regulated and miRNA-17-92 cluster was down-regulated in CRR cells [20,21]. Overexpression of miRNA-17-92 resulted in the loss of radioresistance in CRR cells. However, overexpression of miRNA-17-92 in the parental cells fails to affect cell survival following irradiation [21]. Conversely, down-regulation of miR-7-5p resulted in the loss of radioresistance in CRR cells and the overexpression of miR-7-5p resulted in radioresistance in CRR cells [20]. Additionally, the Fe^2+^ amount is decreased in CRR cells [20]. Intracellular Fe^2+^ reacts with H_2_O_2_ and produces hydroxyl radicals (^•^OH) by Fenton’s reaction and intracellular ^•^OH peroxidize lipid in the plasma membrane [22]. Lipid peroxidation has been reported to lead to cell death, especially ferroptosis [23]. These results strongly suggest that the microRNAs and ROS are involved in chemoresistance and radioresistance.

However, the relationship between microRNA and ROS in CRR cells has not been elucidated yet. Therefore, in this study, we investigated the relationship between microRNA and ROS, focusing on miR-7-5p. We also investigated the ROS signaling pathways under the control of miR-7-5p and tried to show the mechanism of radioresistance that improves the therapeutic effect.

## 2. Results

### 2.1. Knockdown of miR-7-5p Increased ROS in CRR Cells

**Figure 1:**
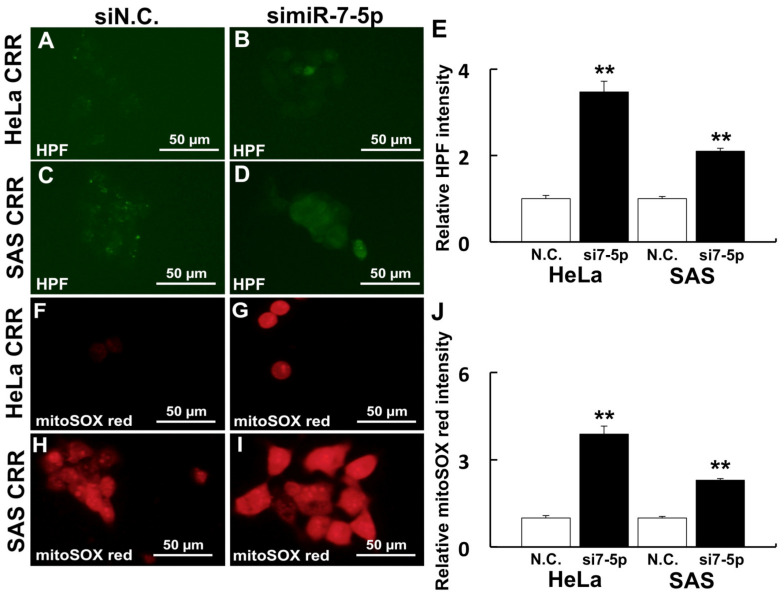
Knockdown of miR-7-5p increased ROS in CRR cells. Cells were treated with 10 µM HPF and 5 µM mitoSOX^TM^ red to detect ^•^OH and mitochondrial superoxide (O_2_^•−^) after a miR-7-5p knockdown, respectively. HPF is a fluorescent probe, which detects hydroxyl radical in cells and mitoSOX^TM^ red is a reagent that emits red fluorescence by reacting with mitochondrial superoxide. (**A**–**E**) Intracellular ^•^OH visualized by HPF. (**F**–**J**) Mitochondrial O_2_^•−^ visualized by mitoSOX^TM^ red. (**A**,**F**) HeLa CRR cells siN.C. treatment. (**B**,**G**) HeLa CRR cells simiR-7-5p treatment. (**C**,**H**) SAS CRR cells siN.C. treatment. (**D**,**I**) SAS CRR cell simiR-7-5p treatment. (**E**) Relative HPF intensity (HeLa CRR N.C.: *n* = 60, HeLa CRR simiR-7-5p: *n* = 30, SAS CRR N.C.: *n* = 32, SAS CRR simiR-7-5p: *n* = 78). (**J**) Relative mitoSOX^TM^ red intensity (HeLa CRR N.C.: *n* = 30, HeLa CRR simiR-7-5p: *n* = 11, SAS CRR N.C.: *n* = 77, SAS CRR simiR-7-5p: *n* = 31). **: *p* < 0.01 using Student’s *t* test. Knockdown of miR-7-5p significantly increased ROS.

**Figure 2:**
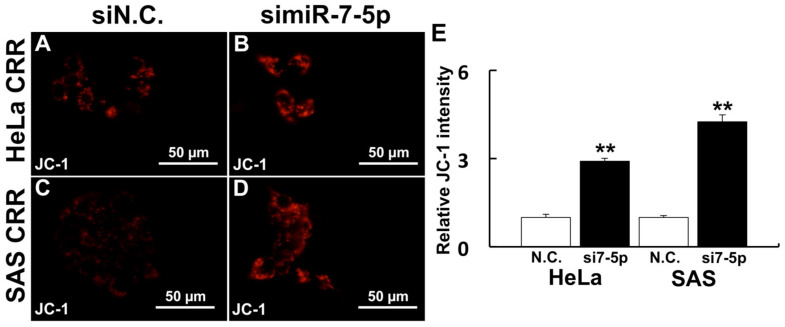
Knockdown of miR-7-5p enhanced ΔΨm. ΔΨm was visualized by 2 µM JC-1 after the miR-7-5p knockdown. JC-1 is a fluorescent probe that is localized mitochondria and is changed fluorescence according to the membrane potential. (**A**) HeLa CRR cells siN.C. treatment. (**B**) HeLa CRR cell simiR-7-5p treatment. (**C**) SAS CRR cells siN.C. treatment. (**D**) SAS CRR cell simiR-7-5p treatment. (**E**) Relative JC-1 intensity (HeLa CRR N.C.: *n* = 11, HeLa CRR simiR-7-5p: *n* = 10, SAS CRR N.C.: *n* = 51, SAS CRR simiR-7-5p: *n* = 25). **: *p* < 0.01 using Student’s *t* test. Knockdown of miR-7-5p significantly increased ΔΨm.

The knockdown of miR-7-5p by siRNA was conducted to investigate the relationship between miR-7-5p and ROS generation. After the knockdown of miR-7-5p in CRR cells, the intracellular ^•^OH amount was increased (Figure 1A–D). Additionally, mitochondrial superoxide generation was also increased by miR-7-5p knockdown (Figure 1E–H). Moreover, ΔΨm was enhanced by miR-7-5p knockdown (Figure 2A–D). These results suggest that miR-7-5p control the ROS generation and change the intracellular organelle status such as mitochondria in CRR cells.

### 2.2. Knockdown of miR-7-5p Enhances Ferroptosis Signaling

**Figure 3:**
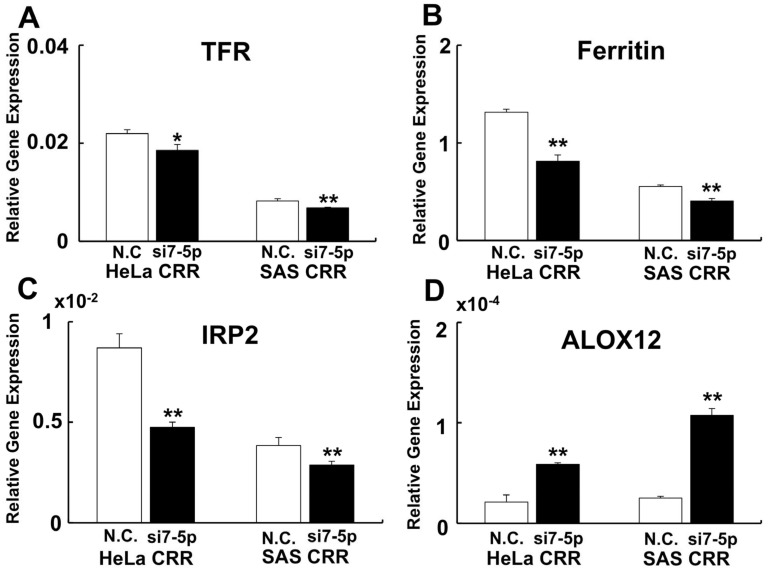
Knockdown of miR-7-5p enhances ferroptosis signaling. To investigate whether ferroptosis signaling was enhanced by knockdown of miR-7-5p, ferroptosis–related gene expressions were performed by qPCR. (**A**) *TFR*. (**B**) *Ferritin*. (**C**) *IRP2*. (**D**) *ALOX12*. MiR-7-5p down-regulate iron-related gene expression and down-regulation of ferritin leads to an increase in intracellular free iron. The expression of *ALOX12*, which enhances ferroptosis, increased after the miR-7-5p knockdown. *: *p* < 0.05, **: *p* < 0.01 using Student’s *t* test. Each qPCR was performed 3 times.

**Figure 4:**
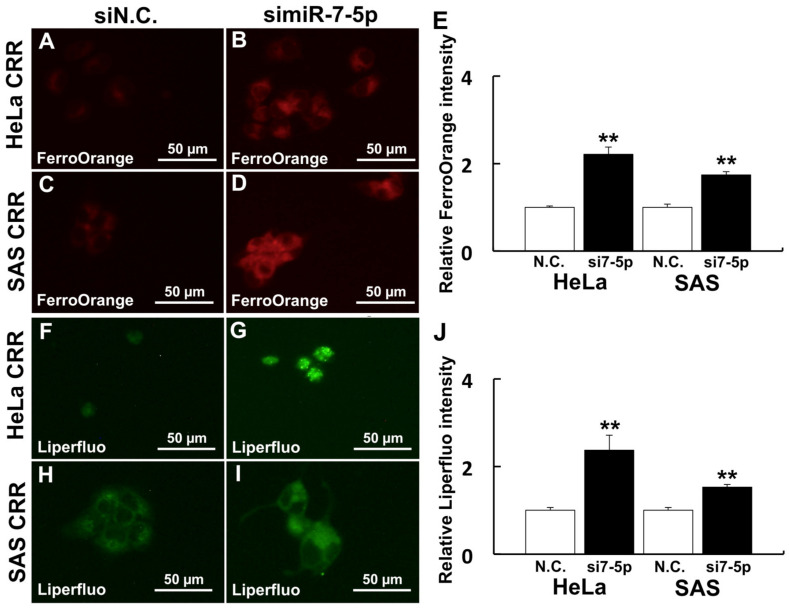
Knockdown of miR-7-5p enhances Fe^2+^ and ferroptosis marker. Cells were treated with 1 µM FerroOrange and 20 µM Liperfluo to detect intracellular Fe^2+^ and lipid peroxidation after a miR-7-5p knockdown. (**A**–**E**) Intracellular Fe^2+^ visualized by FerroOrange. (**F**–**J**) Ferroptosis marker visualized by Liperfluo. (**A**,**F**): HeLa CRR cells siN.C. treatment. (**B**,**G**) HeLa CRR cell simiR-7-5p treatment. (**C**,**H**) SAS CRR cells siN.C. treatment. (**D**,**I**) SAS CRR cells simiR-7-5p treatment. (**E**) Relative FerroOrange intensity (HeLa CRR N.C.: *n* = 41, HeLa CRR simiR-7-5p: *n* = 72, SAS CRR N.C.: *n* = 16, SAS CRR simiR-7-5p: *n* = 63). (**J**) Relative Liperfluo intensity (HeLa CRR N.C.: *n* = 17, HeLa CRR simiR-7-5p: *n* = 12, SAS CRR N.C.: *n* = 32, SAS CRR simiR-7-5p: *n* = 51). **: *p* < 0.01 using Student’s *t*-test. Knockdown of miR-7-5p significantly enhanced Fe^2+^ amount and Liperfluo signal.

To investigate the relationship between miR-7-5p expression and cell death, especially ferroptosis, gene expressions of Fe^2+^-related genes (*Transferrin receptor* [*TFR*], *ferritin*, and *iron responsive protein* [*IRP2*]) and *arachidonate 12-liopxygenase* [*ALOX12*] were examined. As a result, gene expressions of *TFR*, *ferritin*, and *IRP2* were down-regulated when miR-7-5p was knocked down (Figure 3A–C). By contrast, gene expression of *ALOX12* was up-regulated when miR-7-5p was knocked down (Figure 3D). Additionally, the internal Fe^2+^ amount was detected if miR-7-5p controls the internal Fe^2+^ by FerroOrange. As a result, the internal Fe^2+^ amount was increased after miR-7-5p knockdown compared with N.C. (Figure 4A–D). We further analyzed the amount of Liperfluo, a marker of ferroptosis, to examine whether miR-7-5p is involved in ferroptosis after H_2_O_2_ treatment. Knockdown of miR-7-5p enhanced the Liperfluo signal after H_2_O_2_ treatment (Figure 4E–H). These results indicate that the knockdown of miR-7-5p enhances ferroptosis signaling that leads to cell death.

### 2.3. ALOX12 Enhances Intracellular H_2_O_2_ and Lipid Peroxidation

**Figure 5:**
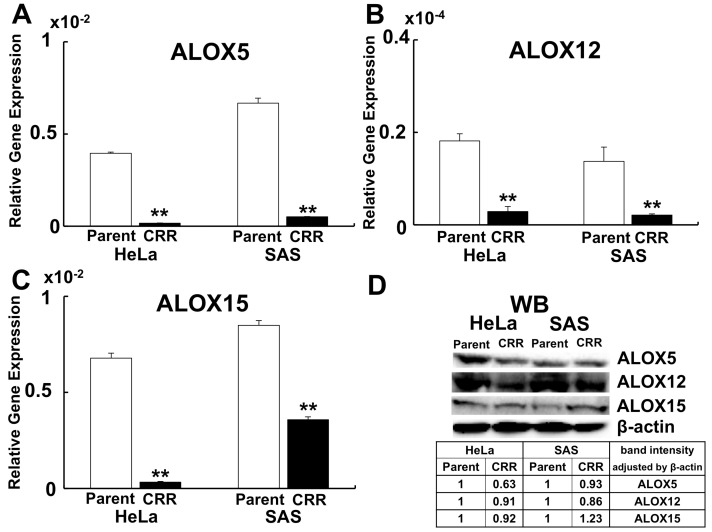
Expression of ALOXs in CRR cells. *ALOX5*, 12, and 15 expressions in parent and CRR cells were detected using qPCR (**A**–**C**) and western blotting (**D**). A: Gene expression of *ALOX5*. (**B**) Gene expression of *ALOX12*. C: Gene expression of *ALOX15*. **: *p* < 0.01 using Student’s *t*-test. Each qPCR was performed 3 times. Gene expressions of *ALOX5*, *12*, and *15* were all significantly down-regulated in CRR cells. (**D**) Western blotting of *ALOX5*, *12*, and *15*. The table shows the expression level of each *ALOX*s in CRR cells. After correcting the intensity of each band by the expression of β-actin, the value is shown with the parent strain as 1. The protein expressions of *ALOX5* and *12* were down-regulated in CRR cells. However, *ALOX15* was not down-regulated in protein level in SAS CRR cells.

**Figure 6:**
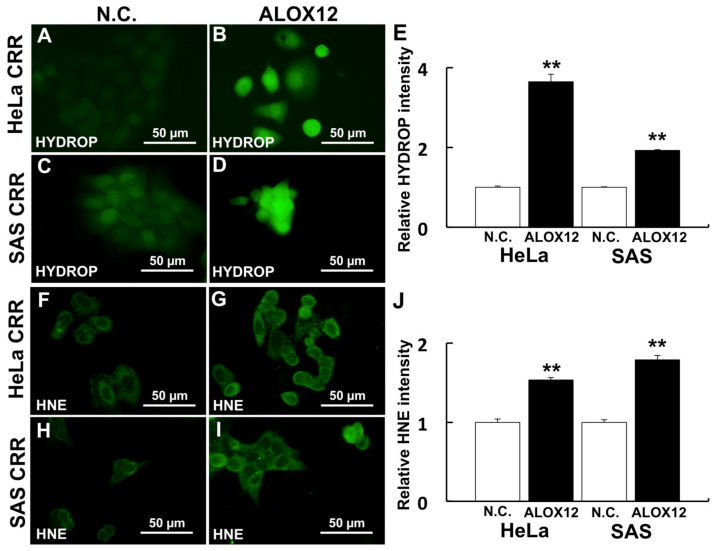
*ALOX12* enhances intracellular ROS and lipid peroxidation. Cells were treated with 2.5 µM HYDROP after 50 µM H_2_O_2_ treatment for 2 h (**A**–**D**) to analyze the effect of *ALOX12* on intracellular ROS generation. Lipid peroxidation was detected using an HNE antibody after 50 µM H_2_O_2_ treatment. (**A**–**E**) Intracellular H_2_O_2_ visualized by HYDROP (**F**–**J**) Lipid peroxidation visualized by HNE antibody. (**A**,**F**) HeLa CRR cells siN.C. treatment. (**B**,**G**) HeLa CRR cells with *ALOX12* overexpression. (**C**,**H**) SAS CRR cells siN.C. treatment. (**D**,**I**) SAS CRR cells with *ALOX12* overexpression. (**E**) Relative HYDROP intensity (HeLa CRR N.C.: *n* = 144, HeLa CRR simiR-7-5p: *n* = 45, SAS CRR N.C.: *n* = 122, SAS CRR simiR-7-5p: *n* = 152). (**J**) Relative HNE intensity (HeLa CRR N.C.: *n* = 58, HeLa CRR simiR-7-5p: *n* = 83, SAS CRR N.C.: *n* = 57, SAS CRR simiR-7-5p: *n* = 87). **: *p* < 0.01 using Student’s *t*-test. *ALOX12* significantly enhances intracellular ROS and lipid peroxidation after H_2_O_2_ treatment.

One of the ferroptosis markers, *ALOX12*, was up-regulated by miR-7-5p (Figure 3D). Conversely, *ALOX12* was down-regulated both at gene expression and protein level (Figure 5A–D). The internal H_2_O_2_ amount and lipid peroxidation were detected using HYDROP and HNE antibody to investigate whether *ALOX12* enhanced ROS generation and lipid peroxidation in CRR cells. HYDROP is a fluorescent probe that specifically detects intracellular H_2_O_2_ and HNE is the by-product of lipid peroxidation and accepted as an oxidative stress. As a result, the overexpression of *ALOX12* enhanced both internal H_2_O_2_ amount and lipid peroxidation after H_2_O_2_ treatment (Figure 6A–H).

### 2.4. MiR-7-5p Affected HIF-1α Expression but Not COX-2 and PFK Expression

**Figure 7:**
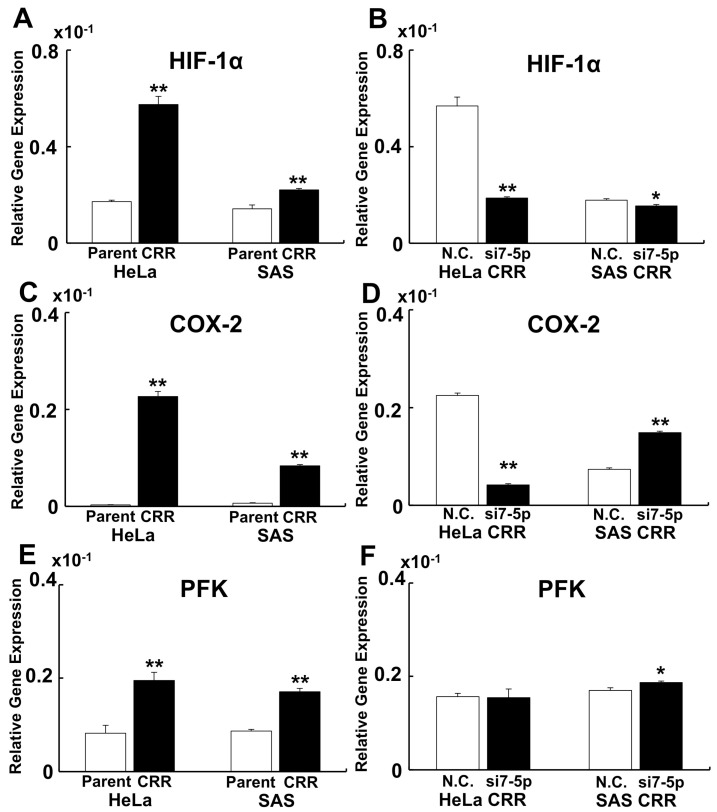
MiR-7-5p affect *HIF-1α* expression but not *COX-2* and *PFK* expression. To reveal whether *HIF-1α*, *COX-2*, and *PFK* are involved in the resistance to radiotherapy and under control of miR-7-5p in CRR cells, qPCR was conducted. (**A**,**B**) Gene expression of *HIF-1α*. (**C**,**D**) Gene expression of *COX-2*. (**E**,**F**) Gene expression of *PFK*. (**A**,**C**,**E**) Gene expression of parent vs. CRR cells. (**B**,**D**,**F**) Gene expression of N.C. vs. miR-7-5p knockdown. *HIF-1α*, *COX-2*, and *PFK* were significantly up-regulated in CRR cells. However, only *HIF-1α* gene expression was regulated by miR-7-5p. *: *p* < 0.05, **: *p* < 0.01 using Student’s *t*-test. Each qPCR was performed 3 times.

To reveal whether Hypoxia Inducible Factor-1α (HIF-1α), *COX-2*, and phosphofructokinase (*PFK*) are involved in the radioresistance and under control of miR-7-5p in CRR cells, qPCR was conducted. As a result, *HIF-1α*, *COX-2*, and *PFK* were significantly up-regulated in CRR cells (Figure 7A,C,E). Knockdown of miR-7-5p down-regulated *HIF-1α* (Figure 7B). However, miR-7-5p knockdown failed to control *COX-2* and *PFK* gene expression (Figure 7D,F).

### 2.5. MiR-7-5p Knockdown Seems to Reverse the Morphology in CRR Cells

**Figure 8:**
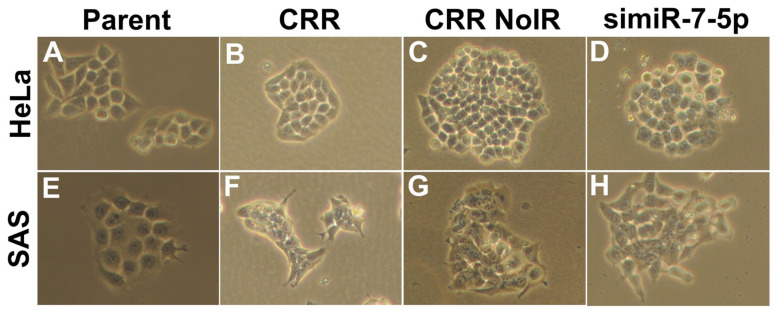
Morphology of parent, CRR, NoIR, and simiR-7-5p cells. Morphology of the cells was observed under the optical microscope with 10× eyepieces and 10× objective lenses (magnification: 100×). (**A**) HeLa parental cells. (**B**) HeLa CRR cells. (**C**) HeLa CRR NoIR cells. (**D**) HeLa CRR cells that were knocked down by miR-7-5p. (**E**) SAS parental cells. (**F**) SAS CRR cells. (**G**) SAS CRR NoIR cells. (**H**) SAS CRR cells that were knocked down miR-7-5p. NoIR cells were cultured without 2 Gy/day irradiation for over 1 year and lost radioresistance. The cell shape of CRR cells looks small and tightly aggregated compared with that in parental cells. These characteristics are similar in NoIR cells, which lose radioresistance. Conversely, when miR-7-5p is knocked down, the cell–cell connections appear to loosen like the parental cells.

We finally investigated whether miR-7-5p affects CRR morphology. We observed parental cells, CRR cells, CRR nonirradiation (NoIR) cells, and miR-7-5p knocked down cells. NoIR cells were cultured without 2 Gy/day irradiation for over 1 year and lost radioresistance. In both HeLa and SAS CRR cells, the cell shape looks small and tightly aggregated compared with parental cells (Figure 8 A,B,E,F). In NoIR cells, which lost radioresistance, the shape is similar to that in CRR cells (Figure 8 C,G). On the other hand, when miR-7-5p is knocked down, the cell–cell connections appear to be loose; the shape is similar to that in parental cells (Figure 8 D,H). The magnification is 100×.

## 3. Discussion

This study shows that miR-7-5p knockdown increased ROS, ΔΨm, and ferroptosis–related signals such as Fe^2+^, *ALOX12*, and *HIF1α* in CRR cells. Furthermore, we also showed that miR-7-5p is involved in the maintenance of cell morphology in CRR cells.

In the previous study, miR-7-5p enhanced radioresistance in CRR cells and in parental cells [20]. Therefore, we believe that miR-7-5p is involved in radioresistance. Several studies reported that miR-7-5p is involved in cancer including chemoresistance [24,25,26]. Yang et al. showed that miR-7-5p promote cisplatin resistance [24]. Wang et al. showed that miR-7-5p inhibits migration and invasion of cancer cells and is involved in epithelial–mesenchymal transformation increasing E-cadherin expression [25]. Song et al. reported that miR-7-5p enhance autophagy via EGFR/AKT/mTOR signaling [26]. In CRR cells, it has been reported that the expression of the EGF receptor and phosphorylation of AKT decreased [20,27], and as reported by Song et al., the mTOR signaling was enhanced [28]. Conversely, it has been reported that the doxorubicin–resistant cell was down-regulated by the miR-7-5p expression [29]. Oxaliplatin resistance cell highly expresses KCNQ1OT1, directly regulated by miR-7-5p [30]. It has been reported that CRR cells that overexpress miR-7-5p did not show resistance to anticancer reagents such as 5-fluorouracil and etoposide [16]. These results suggest that miR-7-5p is considered a very important factor but is not a master control gene in radiation and drug resistance.

Our results show that miR-7-5p decreased ROS and ΔΨm. The main source of intracellular ROS is derived from mitochondria [31]. ΔΨm is generated by proton pumps in mitochondria [32]. These results indicate that the decrease in mitochondrial function occurs in CRR cells. Our previous study demonstrated that CRR cells have low mtDNA and produce less ATP [18]. Furthermore, up-regulation of *PFK*, a glycolysis enzyme, was observed in CRR cells. Low mitochondrial function, i.e., low oxidative phosphorylation, leads to low mtROS production. Low mtROS leads to a decreased intracellular ROS amount, reducing lipid peroxidation. Reduced lipid peroxidation will protect the cell death. Therefore, the mitochondrial condition may be one of the important factors in the resistance of CRR cells.

Recently, the concept of ferroptosis was proposed as a cell death [33,34,35]. Ferroptosis is a cell death via Fe^2+^, ^•^OH, and lipid peroxidation and without caspase activation. Ferroptosis is now one of the effective candidates for cancer treatment because it is becoming clear that other cell deaths such as apoptosis are escaped in cancer cells [36]. A previous study showed that the Fe^2+^, ^•^OH, and lipid peroxidation were decreased in CRR cells [18,20]. In this study, knockdown of miR-7-5p increased Fe^2+^, ^•^OH, and lipid peroxidation in CRR cells. Among iron–related genes, ferritin expression was much higher than TFR and IRP2. The effect of ferritin was to decrease free iron in the cell. Therefore, the decrease in Fe^2+^ was observed in miR-7-5p knockdown cell. Ferrostatin-1, which is an inhibitor of ferroptosis, has been reported recently [37]. Ferrostatin-1 is thought to reduce ROS and inhibit ferroptosis. Further experiments using transfect with miR-7-5p and administration of Ferrostatin-1 would improve our hypothesis.

*ALOX12*, a lipid oxidase, oxidizes plasma membrane lipids and induces ferroptosis [38]. The administration of oxidized lipid resulted in the increase in cell death after H_2_O_2_ treatment [18]. In this study, the expression of *ALOX12* was down-regulated in CRR cells and the expression of *ALOX12* was up-regulated after miR-7-5p transfection. These results indicated that the *ALOX12* is regulated by miR-7-5p. The overexpression of *ALOX12* leads to the increase in internal H_2_O_2_ amount and lipid peroxidation in CRR cells. *ALOX15* gene expression and protein expression were reversed in SAS CRR cell. Gene expression is a quantitative indicator of protein synthesis, but it may not actually correlate. It has been reported that this is due to the existence of a post-transcriptional regulatory mechanism [39]. Since *ALOX15* is also subject to post-transcriptional regulation, there may be no correlation between gene expression and protein expression this time. Further investigation will be needed to clarify the relationship between miR-7-5p and *ALOX15*.

Cancer cells proliferate rapidly, so they cannot reach the bloodstream, resulting in malnutrition and hypoxia. Cancer cells avoid cell death because of malnutrition and hypoxia by enhancing *HIF-1α* expression [40]. It has been shown that up-regulation of *HIF-1α* enhances glycolysis and suppresses oxidative phosphorylation. When oxidative phosphorylation is suppressed, the generation of ROS is decreased, and cell death is suppressed. Furthermore, when HIF-1α enhanced the pentose phosphate pathway, the cells gained radioresistance [10,41]. In CRR cells, expression of *HIF-1α* was increased and miR-7-5p knockdown down-regulated *HIF-1α* expression. These results indicate that the expression of *HIF-1α* regulated by miR-7-5p is an important factor for radioresistance in CRR cells.

The *COX-2* and glycolysis are involved in radioresistance [9,42]. Celecoxib, a selective *COX-2* inhibitor, has been used to enhance radiosensitivity [43]. It has been shown that cancer cells use glycolysis, as we mentioned above. We show that *COX-2* and *PFK* expressions were up-regulated in CRR cells but fail to down-regulate miR-7-5p knockdown. These results show that *COX-2* and *PFK* are involved in radioresistance and will be a target for cancer therapy, but they are not regulated by miR-7-5p. We consider that there are other molecules that regulate radioresistance in addition to miR-7-5p. Further investigation will be needed to understand the relationship between *COX-2* and *PFK* against radioresistance.

The morphology in CRR cells was tightly bound and small. This small cell does not change when maintenance irradiation (MI) is stopped (NoIR). The expression of miR-7-5p is down-regulated in NoIR cells [20]. However, the knockdown of miR-7-5p loosened the adhesion between cells. A similar phenomenon occurs with the expression of *COX-2* and *PFK*. Why the cell adhesion state is different NoIR from simiR-7-5p is still unclear. One possibility is that miR-7-5p has different functions, acute and chronic. The acute effect of miR-7-5p knockdown may loosen the cell–cell junction by downregulating E-cadherin [25] and hypoxia state resolved, and consequently, radioresistance may disappear.

In cancer therapy, special treatment may be required in addition to the usual anticancer drugs or radiation therapy when resistant cells exist. Our results provide several candidates to improve cancer therapy. For example, when *HIF-1α* is enhanced, H_2_O_2_ is administered to the hypoxic part of the cancer tissue to supply oxygen or H_2_O_2_ like KORTUC [44], and activate ferroptosis, administrate iron [18], enhance glycolysis, remove glucose and administrate galactose [45]. However, further investigation will be needed to improve cancer therapy and overcome radio-resistant or chemo-resistant cells.

## 4. Materials and Methods

### 4.1. Cell Culture

The HeLa and SAS human cancer cell lines were obtained from the Cell Resource Center for Biomedical Research, Institute of Development, Aging and Cancer, Tohoku University, Sendai, Japan. HeLa and SAS CRR cells were established by exposing the cells to gradually increasing doses of X-rays (0.5–2 Gy) [17,46]. Cells were cultured in RPMI 1640 (Fujifilm Wako Pure Chemical Corporation, Osaka, Japan) with 10% FBS (Biological Industries, Cromwell, CT, USA) in a humidified atmosphere at 37 °C with 5% CO_2_. CRR cells were cultured in RPMI with 10% FBS without MI for over 1 year to obtain the CRR NoIR cells. All the experiments in this study were conducted using cells in the exponentially growing phase.

### 4.2. Inhibition of miR-7-5p by siRNA Transfection

mirVana^TM^ miRNA Inhibitor (Thermo Fisher Scientific, Waltham, MA, USA), was transfected into CRR cells using Lipofectamine^®^ RNAiMAX Transfection Reagent (Thermo Fisher Scientific) according to the manufacturer’s protocol to inhibit the miR-7-5p expression. mirVana^TM^ miRNA Mimic Negative Control (Thermo Fisher Scientific) was used as a negative control.

### 4.3. Measurement of Intracellular ROS

Intracellular ^•^OH were detected using HPF (hydroxyphenyl fluorescein; Goryo Chemical Inc., Sapporo, Japan) and mtROS were detected using MitoSOX^TM^ (Thermo Fisher Scientific) as recommended by the manufacturer. Briefly, HeLa and SAS CRR cells were cultured overnight in glass bottom dishes (Matsunami Glass Ind., Ltd., Osaka, Japan). Then, the cells were washed twice using Hank’s balanced salt solution (HBSS) (Fujifilm Wako) to remove residual FBS. For ^•^OH, cells were treated with 10 µM of HPF in HBSS for 15 min at 37 °C. For mtROS, cells were treated with 5 µM of MitoSOX^TM^ in HBSS for 10 min at 37 °C. After these treatments, cells were washed with HBSS three times and fluorescence images were obtained using a BZ-8000 fluorescence microscope (KEYENCE Corporation, Osaka, Japan) from 3 separate dishes for each treatment.

### 4.4. Measurement of ΔΨm

ΔΨm was detected using 5,5′,6,6′ tetrachloro 1,1′,3,3′ tetraethylbenzimidazolylcarbocyanine iodide (JC-1: Thermo Fisher Scientific). Cells in the glass bottom dish were treated with 2 µM of JC-1 in RPMI 1640 for 30 min at 37 °C. After these treatments, cells were washed with HBSS three times and fluorescence images were obtained using a BZ-8000 fluorescence microscope from 3 separate dishes for each treatment.

### 4.5. Quantitative PCR (qPCR)

The qPCR was conducted as described previously [47] with slight modification. Total RNA from the cells was extracted using ISOGEN (Nippon Gene Toyama, Japan). After the reverse transcription by ReverTra Ace (TOYOBO CO Ltd., Osaka, Japan), cDNA equivalent to 1 ng total RNA was used for qPCR. The qPCR reactions were conducted by an Applied Biosystems 7300 instrument (Applied Biosystems, Foster City, CA, USA) using THUNDERBIRD^®^ qPCR Mix (TOYOBO). β-actin was used as the loading control. PCR amplification was conducted as follows: one cycle at 95 °C for 10 min, followed by 40 cycles of 95 °C for 10 s and 60 °C for 60 s. Each experiment was conducted 3 times independently. Table 1 shows the primer sequences used in this experiment.

### 4.6. Measurement of Intracellular Fe^2+^ Amount

FerroOrange (Goryo Chemical Inc.) was used to detect intracellular Fe^2+^ as described previously [48]. In the glass bottom dish, cells were treated with 1 μM FerroOrange in HBSS for 30 min at 37 °C. After incubation, FerroOrange was removed by washing three times with HBSS. Fluorescence images were obtained using a BZ-8000 fluorescence microscope from 3 separate dishes for each treatment.

### 4.7. Ferroptosis Detection by Liperfluo after H_2_O_2_ Treatment

Lipid peroxidation after H_2_O_2_ treatment was detected via Liperfluo (Dojindo), the ferroptosis marker. Cells in the glass bottom dish were treated with 50 µM of H_2_O_2_ in RPMI 1640 for 2 h at 37 °C. After removing H_2_O_2_, cells were washed with HBSS three times. Cells were then treated with 10 µM of Liperfluo in HBSS for 30 min at 37 °C. After removing Liperfluo, cells were washed with HBSS three times, and fluorescence images were obtained using a BZ-8000 fluorescence microscope from 3 separate dishes for each treatment.

### 4.8. Western Blotting

Western blotting was conducted as described previously [49]. Briefly, cells were extracted in lysis buffer (50 mM Tris-HCl [pH 7.5], 150 mM sodium chloride, 1% Nonidet P-40, 0.1% sodium deoxycholate, 1 mM sodium fluoride, 1 mM sodium vanadate, and 1 mM PMSF). Each cell lysate (30 µg per lane) was subject to SDS-PAGE under reduced condition using 15% polyacrylamide gel. Proteins were subsequently blotted on a PVDF membrane. After blocking with buffer (3% skim milk in TBS-T; TBS with 0.05% Tween 20, for *ALOX12* antibody and 5% skim milk in PBS-T for the other primary antibodies), the blotted membranes were incubated with primary antibodies (rabbit anti-*ALOX5*, -*ALOX12*, -*ALOX15*: Abcam, Cambridge, UK; ab169755, ab211506, ab80221, dilution factor: 1:1000) at 4 °C overnight. After five washes with TBS-T (*ALOX12* antibody) or PBS-T (other antibodies), the membranes were incubated with peroxidase-conjugated anti-rabbit IgG antibody (#7074; Cell Signaling Technology, Danvers, MA, USA) at room temperature for 2 h. Immunoreactive proteins were visualized with ECL prime (GE Healthcare) using ChemiDoc^TM^ XRS Plus (BIO-RAD Laboratories, Inc., Hercules, CA, USA). Anti-β–actin antibody (NB100-56874; Novus Biologicals LLC, Centennial, CO, USA, dilution factor: 1:1000) was used as a loading control.

### 4.9. Overexpression of ALOX12 and Detection of Internal H2O2 and HNE

For *ALOX12* overexpression, full-length *ALOX12* in pCDNA3 vector (a kind gift from Dr. Torii, Gunma University, Gunma) was used. The plasmid was purified using QIAGEN Plasmid Midi Kit (QIAGEN GmbH, Hilden, Germany). Transfection was conducted using Lipofectamine^®^ 2000 as recommended by the manufacturer (1.6 µg DNA was transfected per 1 mL medium). Internal H_2_O_2_ and HNE, plasma membrane lipid peroxidation, were detected via 2.5 µM HYDROP (Goryo Chemical Inc.) and mouse anti-HNE antibody (Japan Institute for the control of aging, Shizuoka, Japan; 1:200 dilution) as described previously [18]. Fluorescence images were obtained from 3 separate dishes for each treatment.

### 4.10. Statistical Analysis

All the statistical analyses were conducted using Student’s *t*-test. *p* < 0.05 was considered statistically significant. The results are expressed as means ± standard error.

## 5. Conclusions

We showed that miR-7-5p knockdown enhanced ROS amount in CRR cells. Knockdown of miR-7-5p also facilitated ferroptosis signaling such as *ALOX12* expression. Furthermore, overexpression of *ALOX12* enhances intracellular H_2_O_2_ amount and lipid peroxidation. We also demonstrated the relationship between miR-7-5p and *HIF-1α*, *COX-2*, *PFK*, and morphology of the cell. Our results suggest the novel therapeutic strategy to be able to overcome radioresistance decreasing ROS generation by controlling microRNA that regulates ferroptosis.

## Figures and Tables

**Table 1:** Primer sequence used in this study.

Primer Name	Primer Sequence
*TFR* F	5′-ACACGCTGCCAGCTTTACTGGAGAACTT-3′
*TFR* R	5′-AGAGGGCATTTGCAGCTCCCTGAATA-3′
*Ferritin* F	5’-TCTCCTGAAGATGCAAAACCAGCGTG-3’
*Ferritin* R	5’-CAGCTTTCATGGCGTCTGGGGTTTTA-3’
*IRP2* F	5′-GACAAGCACTGGAAAAGTATTCAGCGTGAT-3′
*IRP2* R	5′-TCGTGCCACAAAGTTTAATAATCCTCCATG-3′
*ALOX12* F	5’-TTCAAATGGCCATCTCATGGCATCTGAGT-3’
*ALOX12* R	5’-ATCTGTTCGGAATTGGTTTAGCACAGCTTT-3’
*ALOX5* F	5′-CTGGGCATGTACCCAGAAGAGCATTTTAT-3′
*ALOX5* R	5′-ACAAGTAGTAATATGGCAGCTGCTTCTTCT-3′
*ALOX15* F	5′-ATCTATCGGTATGTGGAAGGAATCGTGAGT-3′
*ALOX15* R	5′-TAAAGAGACAGGAAACCCTCGGTCCT-3′
*HIF-1α* F	5′-AATACCCTCTGATTTAGCATGTAGACTGCT-3′
*HIF-1α* R	5′-TCTGAGTAATTCTTCACCCTGCAGTAGGT-3′
*COX-2* F	5′-TGGAGCACCATTCTCCTTGAAAGGACTTAT-3′
*COX-2* R	5′-GACTGTTTTAATGAGCTCTGGATCTGGAAC-3′
*PFK* F	5′-TGGAGCACATGACGGAGAAGATGAAGAC-3′
*PFK* R	5′-AGTCGAAGACGCCCTTGCCCTCT-3′
*β-actin* F	5′-AGAGCTACGAGCTGCCTGAC-3′
*β-actin* R	5′-AGCACTGTGTTGGCGTACAG-3′

## Data Availability

The data presented in this study are available on request from the corresponding author. The data are not publicly available since they are raw data.
